# Metabolic Perturbation and Potential Markers in Patients with Esophageal Cancer

**DOI:** 10.1155/2017/5469597

**Published:** 2017-04-20

**Authors:** Xianlan Zhu, Kun Wang, Gaoshuang Liu, Yuqing Wang, Jin Xu, Linsheng Liu, Mengjie Li, Jian Shi, Jiye Aa, Lianzhen Yu

**Affiliations:** ^1^Department of Gastroenterology, The First Affiliated Hospital of Nanjing Medical University, 300 Guangzhou Road, Nanjing 210029, China; ^2^Department of Gastroenterology, Wuxi People's Hospital Affiliated to Nanjing Medical University, 299 Qingyang Road, Wuxi 214023, China; ^3^Department of Gastroenterology, The First People's Hospital of Lianyungang, 182 North Tongguan Road, Lianyungang 222002, China; ^4^Lab of Metabolomics, Key Laboratory of Drug Metabolism and Pharmacokinetics, State Key Laboratory of Natural Medicines, China Pharmaceutical University, 24 Tongjia Avenue, Gulou District, Nanjing 210009, China

## Abstract

Clinical diagnosis of esophageal cancer (EC) at early stage is rather difficult. This study aimed to profile the molecules in serum and tissue and identify potential biomarkers in patients with EC. A total of 64 volunteers were recruited, and 83 samples (24 EC serum samples, 21 serum controls, 19 paired EC tissues, and corresponding tumor-adjacent tissues) were analyzed. The gas chromatography time-of-flight mass spectrometry (GC/TOF-MS) was employed, and principal component analysis was used to reveal the discriminatory metabolites and identify the candidate markers of EC. A total of 41 in serum and 36 identified compounds in tissues were relevant to the malignant prognosis. A marked metabolic reprogramming of EC was observed, including enhanced anaerobic glycolysis and glutaminolysis, inhibited tricarboxylic acid (TCA) cycle, and altered lipid metabolism and amino acid turnover. Based on the potential markers of glucose, glutamic acid, lactic acid, and cholesterol, the receiver operating characteristic (ROC) curves indicated good diagnosis and prognosis of EC. EC patients showed distinct reprogrammed metabolism involved in glycolysis, TCA cycle, glutaminolysis, and fatty acid metabolism. The pivotal molecules in the metabolic pathways were suggested as the potential markers to facilitate the early diagnosis of human EC.

## 1. Introduction

Esophageal cancer (EC) is the eighth most common malignancy globally and the fourth leading cause of cancer mortality in China [1]. Although surgical resection remains the first choice of curative treatment for localized cancer, treatment outcomes and 5-year survival rates are far from satisfactory, because the early symptoms of EC are insidious and most patients present with incurable disease [2, 3]. Therefore, more accurate and robust biomarkers are in great demand for early screening and diagnosis of EC.

Metabolomic analysis is a systemic tool focusing on endogenous low molecular weight compounds to quantitatively assess metabolic features and has been shown to be effective for elucidation of biomarkers, metabolic pathways, and disease diagnosis [4–7]. Metabolic reprogramming has been widely observed in various tumors [8–11]. Metabolomics increases the possibility of validation of candidate biomarkers in the prospective studies through an accurate screening process for marker identification [12–14]. This approach also enhances the ability of researchers to analyze metabolomic data of specific biomarkers to gain insight into disease biology [15]. Previous studies have applied nuclear magnetic resonance (NMR) spectrometry and gas chromatography/mass spectrometry (GC/MS) [16, 17] to profile metabolites in serum, urine, and tissue of EC separately and respectively. Jin et al. [18] identified metabolomic signatures in serum of patients with lymph node metastasis based on GC/MS, while Davis et al. [19] used NMR spectroscopy to profile metabolic phenotypes of urine in EC and Barrett's esophagus. Although these studies identified panels of discriminant metabolites and suggested potential markers, the metabolites were case dependent and not correlated to the perturbed metabolites in EC tissues.

Hence, the current study was designed to profile metabolites in both serum and tissue samples from clinical patients with primary EC based on gas chromatography time-of-flight mass spectrometry (GC/TOF-MS). We aimed to screen potential markers that were characterized in serum and EC tissues and evaluate the sensitivity and specificity of potential markers for EC diagnosis.

## 2. Materials and Methods

The study protocol was approved by the Ethics Committee of Nanjing Medical University, and informed consent was obtained from all patients and volunteers. The inclusion and exclusion criterions in this study were assessed based on the entire body. Inclusion criterions were (i) age > 18 years and no prior chemotherapy or radiotherapy before enrollment and (ii) definite pathological diagnosis. Exclusion criterions were (i) a comorbidity of a metabolic disease, such as diabetes mellitus, gout, hyperlipidemia, or hemopathy; (ii) pregnancy or lactation; (iii) any symptom of massive stress or acute disease in the previous 2 weeks, such as large area of burns, psychic trauma, fever, cough, vomiting, and diarrhea; and (iv) use of specific drugs during the last 2 weeks, such as antibiotics, hormones, or nonsteroid anti-inflammatory drugs.

Serum samples were collected from 21 volunteers and 24 EC patients under fasting conditions. Tissue samples were collected from an additional 19 EC patients. Primary EC tissue specimens were excised from the central no-necrotic zone to ensure harvesting of cancer cells. Tumor-adjacent tissues were excised 2–3 cm from the tumor margin. All tissue samples were immediately snap-frozen in liquid nitrogen and stored at −80°C until assayed. Serum samples were collected in ethylenediaminetetraacetic sodium anticoagulated tubes and then centrifuged at 4000*g* for 10 min. At least 500 *μ*L of serum was collected and frozen at −80°C until assayed. Detailed information regarding reagents, sample preparation, and GC/TOF-MS analysis is provided in the Supplementary Materials available online at https://doi.org/10.1155/2017/5469597.

## 3. Results

### 3.1. Study Population

The clinical characteristics of the patients and volunteers are summarized (Table 1). There were no significant differences in gender, age, and body weight between the EC patients and healthy controls (*P* > 0.05). The vast majority (90.70%) of patients had ESCC of histological grades I–III. Most patients (95.35%) had cancer in the middle or lower thoracic or esophagogastric junction, and almost half (48.84%) had lymph node metastasis. Of all patients, 36 consented to computer tomography (CT) examinations, yet three of the 36 CT examinations revealed no lesions. Besides, most patients had normal hepatic and renal function.

### 3.2. Metabolic Phenotypes of EC

**Figure 1:**
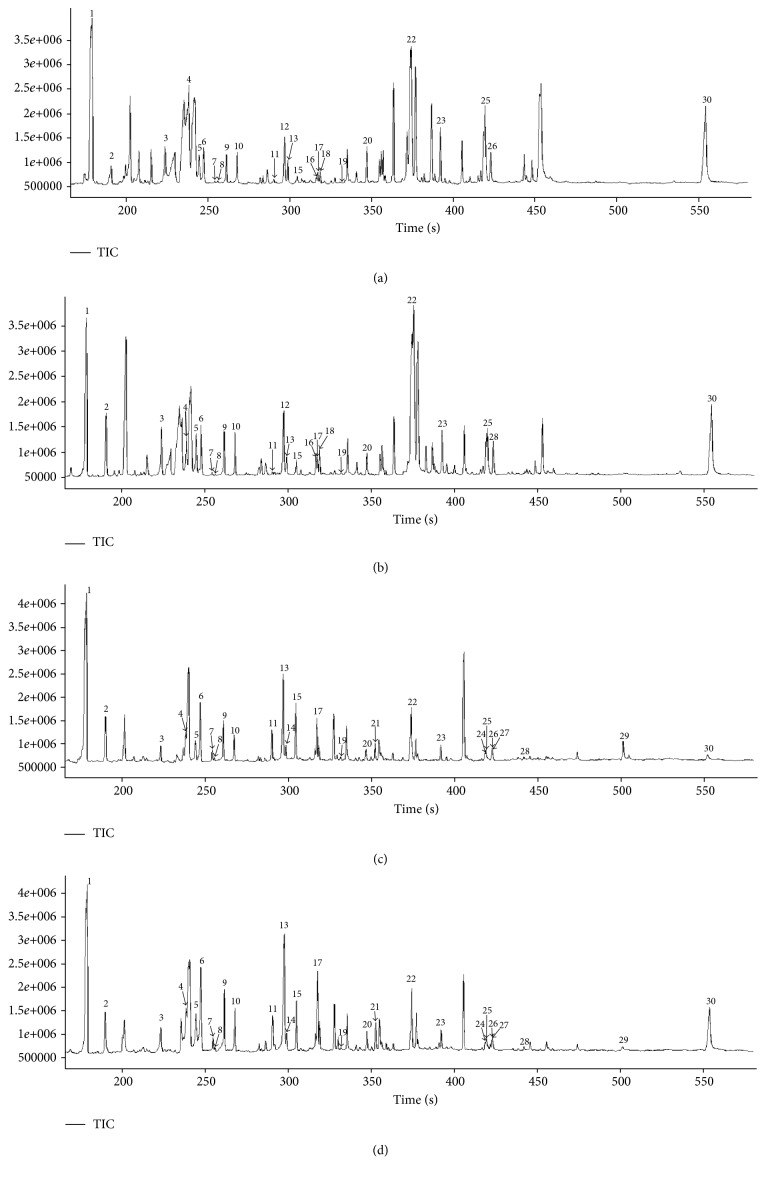
Representative GC/TOF-MS total ion current (TIC) chromatograms of serums and tissues: (a) patient serum, (b) normal serum, (c) cancer tissue, and (d) tumor-adjacent tissue. Parts of the peaks were identified as 1, lactic acid; 2, alanine; 3, valine; 4, leucine; 5, proline; 6, glycine; 7, uracil; 8, fumaric acid; 9, serine; 10, threonine; 11, malic acid; 12, pyroglutamic acid; 13, asparagine; 14, hydroxyproline; 15, creatinine; 16, ornithine; 17, glutamic acid; 18, phenylalanine; 19, ribose; 20, glutamine; 21, hypoxanthine; 22, glucose; 23, hexadecanoic acid; 24, linoleic acid; 25, oleic acid; 26, tryptophan; 27, stearic acid; 28, glucose-6-phosphate; 29, maltose; and 30, cholesterol.

**Figure 2:**
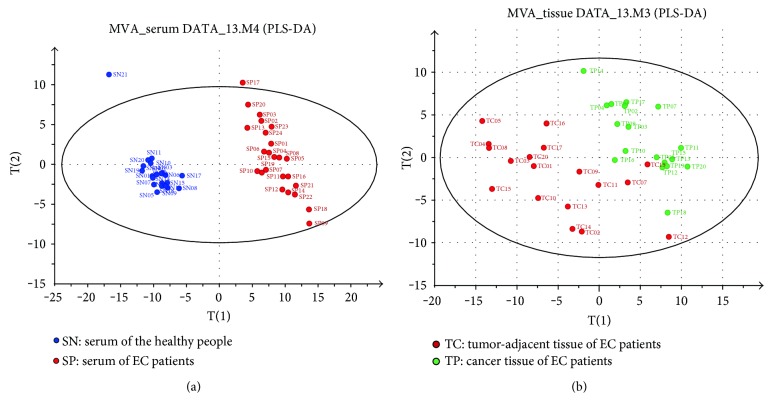
The projection to latent structures and discriminant analysis score plot. (a) SN, normal serum; SP, patient serum. The two groups of serum were completely scattered into two different regions. There were two samples on the outside of the oval, which were far away from the others. It indicated that the metabolic profilings of the two people were significantly different from others, and we had eliminated the two samples when performing two sample *t*-tests. (b) TC, tumor-adjacent tissue; TP, cancer tissue.

Representative total ion current chromatograms are presented in Figure 1. A total of 61 metabolites in serum and 58 in tissues were identified, which included carbohydrates, amino acids, organic acids, and fatty acids. Multivariate statistical analysis was performed using PLS-DA, and each dot in the score plot represented a sample. The scattering of samples in the score plots was exclusively dependent on the composition and concentration of the molecules in each sample. As shown in Figures 2(a) and 2(b), serum samples and cancer tissues were well separated with the controls located in two different regions. The higher explicative and predictive capacities of serum (*R*^2^*Y* = 98%, *Q*^2^*Y* = 95%) suggested differences in most of the detected variable molecules in serum between the EC patients and controls. The relatively lower *R*^2^*Y* (76.4%) and *Q*^2^*Y* (46.0%) values of tissues indicated that the differences in molecular compositions between the EC tissues and the tumor-adjacent tissues were not as great as those in serum, although still significant.

### 3.3. Distinct Metabolites of EC Patients

Statistical analysis revealed that 42 compounds in serum and 37 in tissues were the discriminant molecules between the EC patients and controls (Table 2). In general, the concentrations of molecules in serum and tissues of the EC patients were diverse. The pivotal metabolites in glycolysis, pyruvic acid and lactic acid, showed a reverse tendency, while the level of glucose was low in both serum and tissues of the EC group. The majority of intermediates in the tricarboxylic acid (TCA) cycle (citric acid, *α*-ketoglutarate, fumaric acid, and malic acid) were elevated in both serum and tissue samples from the EC patients. Most concentrations of amino acids were lower in serum from the EC patients as compared with those from the controls, but at greater levels in cancer tissues than normal tumor-adjacent tissues. Interestingly, we found that the concentrations of most fatty acids were lower in serum collected from the EC patients but showed a reverse tendency in tissues. The levels of cholesterol, myo-inositol-1-phosphate, uracil, and hypoxanthine were higher in both serum and tissues of the EC patients than those of the controls.

### 3.4. Diagnostic Value of Potential Biomarkers of EC

**Figure 3:**
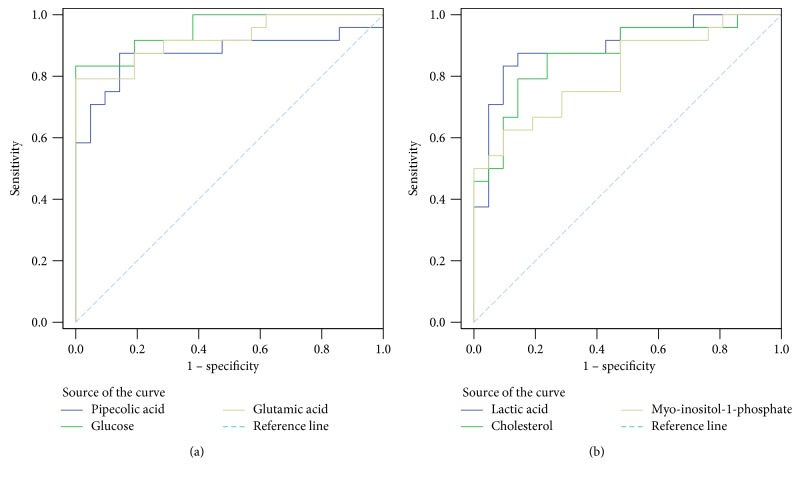
ROC curve analysis of potential serum biomarker levels. (a) Low levels of biomarkers in the EC group. (b) High levels of biomarkers in the EC group.

To characterize the EC patients from the healthy controls, receiver operating characteristic (ROC) curve analysis of potential biomarkers in serum was performed (Figures 3(a) and 3(b)), and cutoff values were calculated according to the Youden indices, sensitivities, specificities, and AUC values. For example, the cutoff value of glucose was 109.60, with a sensitivity of 83.3%, specificity of 100.0%, and AUC value of 0.952 (95% confidence interval = 0.897–1.000) (Tables 3 and 4). The ROC curves indicated that these markers had good diagnostic and prognostic values for EC.

## 4. Discussion

**Figure 4:**
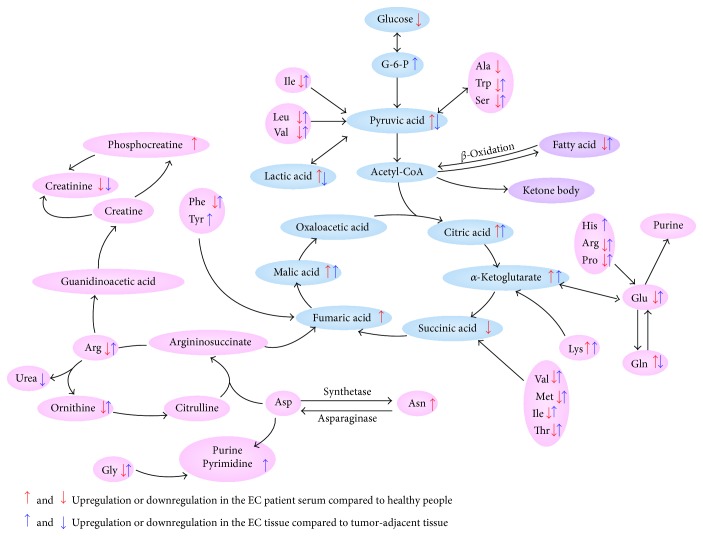
Map illustrating the main metabolic pathways for the most relevant distinguishing metabolites. G-6-P, glucose-6-phosphate; Ala, alanine; Trp, tryptophan; Ser, serine; Ile, isoleucine; Leu, leucine; Val, valine; His, histidine; Arg, arginine; Pro, proline; Glu, glutamic acid; Gln, glutamine; Lys, lysine; Met, methionine; Thr, threonine; Asp, aspartic acid; Asn, asparagine; Phe, phenylalanine; Tyr, tyrosine; and Gly, glycine.

It is well documented that metabolism reprogramming occurs in various cancer cells [8, 20, 21]. According to the discriminant molecules in this study, most metabolic pathways were perturbed in the EC patients, at both systemic (peripheral blood) and local (EC tissues) levels (Figure 4). Measurement of these molecules has the potential to identify candidate markers suggestive of reprogrammed metabolism [22].

The Warburg effect is a process that cancer cells prefer anaerobic glycolysis rather than oxidative phosphorylation for energy production, even when provided with sufficient oxygen [23]. Apart from previous research, serum and tissues as a whole and ROC curve analysis in the present study identified glucose and lactic acid concentrations as potential biomarkers of EC. The low level of glucose in both EC serum and tissues suggested that cancer cells acquire large quantities of glucose, which accelerates lactic acid accumulation in serum via glycolysis. Lactic acid and pyruvate, which were increased in serum, but decreased in tissues of the EC patients, revealed enhanced gluconeogenesis of EC cells, consistent with gastric cancer [24]. Lactic acid in serum and cancer tissues may indicate poor prognosis in many types of cancers [25–27]. In this study, most intermediates of the TCA cycle accumulated both in EC serum and tissues. Most cancer cells were always under anoxic conditions due to accelerated proliferation, as compared with normal cells. So, the process of oxidative phosphorylation was limited and the metabolic pathway was impeded, which was a well-known barrier-like effect. This disruption in the TCA cycle further verified the presence of enhanced glycolysis.

Glutamine and glutamic acid play important roles in cancer cell proliferation. Glutamic acid is an important energy source via the TCA cycle after conversion to *α*-ketoglutarate [28]. Besides glutamic acid, glutamine contributes to de novo fatty acid synthesis and serves as a nitrogen source [29, 30]. In this study, glutamine in EC tissues was excessively consumed for the proliferation of cancer cells, resulting in decreased tissue concentrations. The intensive metabolism of glutamine suggested that EC cells require large amounts of glutamic acid from the systemic environment, resulting in decreased serum glutamic acid levels. The results of the present study demonstrated a significant increase in uracil content in EC tissues, which further confirmed that glutaminolysis was an indispensable metabolic pathway of de novo synthesis of purine and pyrimidine bases.

In this study, the level of most amino acids, such as tryptophan, serine, isoleucine, leucine, and valine, decreased in EC serum and increased in tissues. These amino acids participated in gluconeogenesis and energy production, which led to the large depletion of these amino acids from serum. In addition to energy production, serine, tryptophan, and glycine supply one carbon unit for synthesis of purines and pyrimidines. The branched-chain amino acids leucine, isoleucine, and valine are used as nitrogen sources by cancer cells [18]. Because of the rapid proliferation of cancer cells, copious amounts of asparagine were absorbed by the cancer tissues for production of hereditary material, which led to increased asparagine levels in EC tissues. In this study, we found decreased EC serum and increased tissue tryptophan levels, along with the elevated kynurenic acid concentrations in EC tissues, suggesting an enhanced cancer cell immune escape in EC [31].

In the present study, the EC patients had significantly higher cholesterol levels than the healthy controls. Excessive amounts of nonesterified free cholesterol are required to meet the metabolic requirements of rapidly proliferating cancer cells, which also tend to accelerate the release of free cholesterol from esterified cholesterol. The high cholesterol level in EC serum was in contrast to the findings of previous studies [19]. ROC curve analysis identified cholesterol as a potential biomarker of EC. Generally, concentrations of most free fatty acids (FFAs) are increased in EC serum but decreased in cancer tissues. We presume that the EC cells take in just enough FFAs to promote the release of fatty glyceride. In fact, the depressed 2-hydroxyvaleric levels in EC serum suggested inhibition of *β*-oxidation of FFAs.

The EC patients showed distinct reprogrammed metabolism involved in glycolysis, glutaminolysis, and TCA synthesis. Although the abundances of these discriminatory metabolites in serum and tissue were not always positively correlated, the metabolic phenotype of serum in the EC patients was closely associated with that of tissues. Future work involving larger populations of patients with EC should be performed to confirm our findings, and more studies are encouraged to investigate the influence of the lymph node metastasis on metabolic perturbation.

## Supplementary Material

Supplementary Materials are detailed descriptions of reagents, sample preparation and GC/TOF-MS analysis.

## Figures and Tables

**Table 1:** Patients' characteristics in the study of esophageal cancer.

Characteristics	Patients supplying serum	*P*_1_	Patients supplying tissue	*P*_2_	Healthy control
Gender (*n*, male/female)	19/5	1.000	15/4	1.000	17/4
Age [mean (range)]	60.2 (48–86)	0.960	65.4 (44–87)	0.122	60.0 (45–86)
Weight [mean (range)]	61.6 (42–78)	0.597	62.4 (42–79)	0.895	62.8 (47–75)
Histology (*n*)					
ESCC	22	—	17	—	—
Adenocarcinoma	2	—	2	—	—
Histologic grade (*n*)					
I	6	—	2	—	—
II	8	—	10	—	—
III	10	—	7	—	—
Location (*n*)					
Cervical	0	—	0	—	—
Upper thoracic	1	—	1	—	—
Middle thoracic	11	—	7	—	—
Lower thoracic/EGJ	12	—	11	—	—
Lymph node metastasis (*n*)					
Yes	13	—	8	—	—
No	11	—	11	—	—
CT (*n*/*n*)	17/20	—	16/16	—	—
ALT [mean ± SD]	24.87 ± 35.99	—	17.63 ± 6.18	—	—
Creatinine^a^ [mean ± SD]	78.21 ± 24.34	—	79.79 ± 27.50	—	—

^a^Serum creatinine; EGJ: esophagogastric junction; CT: computer tomography; ALT: alanine transaminase; SD: standard deviation; *P*_1_: *P* value of the EC patients supplying serum compared with the healthy control; *P*_2_: *P* value of the EC patients supplying tissue compared with the healthy control.

**Table 2:** The variation trend of metabolites in serum or tissue compared to the control.

Metabolites	The regulatory tendency and statistical analysis relative to the controls			
Groups	Serum		Tissue	
Glycolysis				
Glucose	↓↓	^∗^	↓	/
Pyruvic acid, lactic acid	↑↑	^∗^	↓	^∗^
Glucose-6-phosphate	±	/	↑↑	^∗^
Ribose	↓↓	^∗^	↑↑	^∗^
TCA cycle				
Citric acid, *α*-ketoglutarate, and malic acid	↑↑	^∗^	↑↑	^∗^
*cis*-Aconitic acid	↑	^∗^	±	/
Succinic acid	↓	^∗^	↑↑	/
Fumaric acid	↑	^∗^	↑	/
Amino acid metabolism				
Arginine	↓↓	^∗^	↑	^∗^
Glutamic acid, glycine, and phenylalanine	↓↓	^∗^	↑↑	^∗^
Tryptophan, methionine	↓	^∗^	↑	^∗^
Serine, isoleucine, leucine, valine, proline, threonine, hydroxyproline, and ornithine	↓	^∗^	↑↑	^∗^
Tyrosine, kynurenic acid, asparagine, and histidine	±	/	↑↑	^∗^
Alanine	↓	^∗^	↑	/
Cystine	↓↓	^∗^	↑↑	/
Taurine	↑↑	^∗^	±	/
Pyroglutamic acid, creatinine	↓	^∗^	↓	^∗^
Urea	±	/	↓↓	^∗^
Urate	±	/	↑	^∗^
Lysine	↑	^∗^	↑	^∗^
Cysteine	↑	^∗^	↑	/
Glutamine	↑↑	^∗^	↓	^∗^
Fatty acid metabolism				
Monostearin, pipecolic acid	↓↓	^∗^	±	/
Tetradecanoic acid, glyceric acid, docosahexaenoic acid, hexadecanoic acid, and 2-hydroxyvaleric acid	↓	^∗^	±	/
Stearic acid	±	/	↑	^∗^
Oleic acid, aminomalonic acid	±	/	↑↑	^∗^
Cholesterol, myo-inositol-1-phosphate	↑	^∗^	↑	/
Linoleic acid	↓	^∗^	↑	^∗^
Nucleotide metabolism				
Uracil	±	/	↑↑	^∗^
Hypoxanthine	↑↑	/	↑	^∗^

↓, ↓↓ show downregulation by at least 20% or 50%, respectively.

↑, ↑↑ show upregulation by at least 20% or 50%, respectively.

± shows marginal regulation without statistical significance; ∗ shows *P* < 0.05; / shows *P* > 0.05.

**Table 3:** ROC curves of low-level biomarkers in the EC group.

Marker	Cutoff value^a^	Sensitivity	Specificity	AUC	*P* value	95% CI^b^
Pipecolic acid	8.50	87.5%	85.7%	0.875	<0.001	0.762–0.988
Glucose	109.60	83.3%	100.0%	0.952	<0.001	0.897–1.000
Glutamic acid	27.44	79.2%	100.0%	0.923	<0.001	0.844–1.000
Oleic acid	14.62	83.3%	66.7%	0.794	0.001	0.663–0.924

^a^Cutoff value (×10^5^); ^b^95% confidence interval of the difference.

**Table 4:** ROC curves of high-level biomarkers in the EC group.

Marker	Cutoff value^a^	Sensitivity	Specificity	AUC	*P* value	95% CI^b^
Lactic acid	661.68	83.3%	90.5%	0.899	<0.001	0.805–0.992
Cholesterol	695.62	79.2%	85.7%	0.869	<0.001	0.764–0.974
Myo-inositol-1-phosphate	6.19	62.5%	90.5%	0.813	<0.001	0.690–0.937

^a^Cutoff value (×10^5^); ^b^95% confidence interval of the difference.
