# Supplementary material for: Metabolic Perturbation and Potential Markers in Patients with Esophageal Cancer
Source: Gastroenterol Res Pract. 2017 Apr 20;2017:5469597. doi: 10.1155/2017/5469597 (PMC5415862; doi:10.1155/2017/5469597)
Supplement: Supplementary file 1 — Supplementary Materials are detailed descriptions of reagents, sample preparation and GC/TOF-MS analysis. [file 5469597.f1.pdf]

TEXT SUPPLYMENTS

Reagents, sample preparation and GC/TOF-MS analysis

Chemicals and reagents

The following chemicals and reagents were used in the present study: N-methyl-N-trimethylsilyl trifluoroacetamide (MSTFA) with 1% trimethylchlorosilane (TMCS; Pierce Biotechnology, Rockford, IL, USA), 98% methoxamine hydrochloride (Sigma-Aldrich Chemie Gmbh, Munich, Germany), standard alkane solution (C_8-C_{40}; Sigma-Aldrich Chemie GmbH, Buchs, Switzerland), pyridine (≥99.8% GC; Sigma-Aldrich, Bangalore, India), stable, isotope-labeled, internal standard (IS) compound ([$^{13}C_2$]-myristic acid; Isotec, Lawrenceville, NJ, USA), methanol (Tedia Co., Inc., Fairfield, OH, USA), n-heptane (Merck KGaA, Darmstadt, Germany), and purified water produced with a Milli-Q system (EMD Millipore, Billerica, MA, USA).

Sample preparation and GC/TOF-MS analysis

Samples were thawed in a 37°C water bath for 15 min. Then, 100 μL of supernatant of centrifuged blood was transferred to an Eppendorf tube and about 20 mg of tissue was weighed. A monophasic mixture of 400 μL of water:methanol (1:4, v:v) containing [$^{13}C_2$]-myristic acid as an internal standard (2.5 μg/mL) was added to the serum sample, which was vortexed for 2 min, cooled at 4°C for 1 h, and then centrifuged at 20,000 rpm for 10 min. Afterward, 100 μL of supernatant was dried in a SpeedVac nitrogen evaporator (Savant Instruments, Inc., Farmingdale, NY, USA) and 30 μL of methoxyamine pyridine solution (10 μg/mL) was added. Then the mixture was vortexed for 3 min and incubated for 16 h at 20°C for methoximation of alcohols. Afterward, 30 μL of MSTFA with 1% TMCS were added for trimethylsilylation. At last, 30 μL of n-heptane was added to 30 μg/mL of methyl stearate and the solution

was vortexed for 3 min for GC/TOF-MS analysis. Meanwhile, 20 mg of tissue was carefully homogenized in 800 µL (1:40, w:v) of monophasic solvent. After extraction and trimethylsilylation, according to the preparation methods used for the serum samples, 30 µL of n-heptane were added and the solution was vortexed for 3 min before analysis.

Each 0.5-µL aliquot of the derivate was injected into a splitless port of an Agilent 6890 gas chromatography system equipped with a 10 m × 0.18 mm i.d. fused-silica capillary column with 0.18 µm DB-5 stationary phase (J&W Scientific, Folsom, CA, USA) using an Agilent 7683 Series autosampler (Agilent Technologies, Atlanta, GA, USA). The inlet temperature was at 250°C and helium was the carrier gas at a constant flow rate of 1.0 mL/min. The flow time was 60 s and the equilibrium time was 1 min. The initial column temperature was held at 70°C for 2 min and then increased to 310°C at a rate of 30°C/min, while holding each stage for 2 min. The column effluent was introduced into the ion source of a Pegasus III mass spectrometer (LECO Corp., St. Joseph, MI, USA). The delivering tube temperature was 250°C and the ion source temperature was 200°C. Mass spectra were acquired in the full scan mode from an m/z ratio of 50 to 800 at a rate of 20 spectra/s, and the delay time of solution was 170 s and the detecting voltage was –1650 V. Mass fragmentation was performed in electron impact (EI) ionization mode at 70 eV with a current of 2.0 mA.

Data processing and statistical analysis

ChromaTOF 2.00 software (LECO Corporation) was used to automatically detect peak values and calculate peak areas. The peak width in automatic peak detection and mass spectrum deconvolution were set at 2 s and peaks of the signal-to-noise ratio less than 20 were rejected. The retention indices of the peaks were calculated based on the

ratio of the retention time and those of the C_8-C_{40} alkane series. For identification, the mass spectra and retention indices of themetabolites were searched in the National Institute of Standards and Technology library 2.0 (2012) Wiley 9 (Wiley-VCH Verlag GmbH & Co. KGaA, Weinheim, Germany), and the discriminant metabolites were further identified with their reference standards respectively. Multivariate data analysis was performed using SIMCA-P 11 software (MKS Umetrics AB, Umeå, Sweden). A data matrix was constructed with the experimental sample names as observational variables and the peak areas normalized to internal standard served as the response variables. Dimension reduction was performed using principal component analysis and partial least squares discriminant analysis (PLS-DA). The maximum differences among groups or samples with several principal components were calculated. A plot of PLS-DA sample scores was used to identify differences among groups. The PLS-DA loading plot was applied to screen the main different endogenous metabolites with variable importance in the projection values of greater than 1. Statistical analysis was performed using the two sample *t*-test and probability (P) value < 0.05 and < 0.001 were considered statistically and very statistically significant. Variables with striking deviations were further evaluated as potential biomarkers and their structures were confirmed by comparison to authentic reference standards.
